# Abrasion of the Teeth

**Published:** 1848-04

**Authors:** J. Taft

**Affiliations:** Surgeon Dentist, Xenia, Ohio.


### ARTICLE IV.

*Abrasion of the Teeth.* By J. TAFT, Surgeon Dentist, Xenia, Ohio.

ON the 5th of November, 1846, Mr. M., aged about 30 years, desired me to examine his teeth. I found some four or five of the molares slightly decayed, which I filled; when directing my attention more particularly to the anterior teeth, I found the superior incisores worn down almost to the necks; the central incisores more than the lateral, and the canines less than the latter. I directed him to close the jaws, when I found the points of the superior and inferior incisores could not be brought nearer together than one-fourth of an inch, by any motion of which the jaw was capable; when the jaws were closed the inferior incisores were almost perpendicular below the superior, at which time the space between the superior and inferior incisores presented a semi-elliptical form, its greatest width being one-fourth of an inch between the superior and inferior central incisores, and its length terminated at the first bicuspides of each side. I inquired of the gentleman if any thing had been used between the teeth which could have worn them to such an extent, when he assured me that nothing of the kind had ever been used, and that the teeth had not been used in mastication, or even for seizing any thing for a number of years. The loss of substance was an entire mystery and wonder to himself. The circle described upon the edges of the superior incisores is as regular as could be drawn with a compass. The inferior incisores have suffered no loss. Had it not been for a beautiful provision of nature, long previous to the present examination, the nerves of the incisores must have been exposed; but there had been a deposit of bony matter, which so completely filled the dental cavities, that the surface presented was as smooth as if it had been burnished. That portion which filled the dental cavity was semi-pellucid. As to the cause, I am not able even to give a reasonable conjecture.

